# Flexible Gaussian Accelerated Molecular Dynamics to Enhance Biological Sampling

**DOI:** 10.1021/acs.jctc.3c00619

**Published:** 2023-08-31

**Authors:** Oriol Gracia Carmona, Chris Oostenbrink

**Affiliations:** † Institute for Molecular Modeling and Simulation, Department of Material Sciences and Process Engineering, University of Natural Resources and Life Sciences, Vienna. Muthgasse 18, 1190 Vienna, Austria; ‡ Christian Doppler Laboratory for Molecular Informatics in the Biosciences, University of Natural Resources and Life Sciences, Vienna. Muthgasse 18, 1190 Vienna, Austria

## Abstract

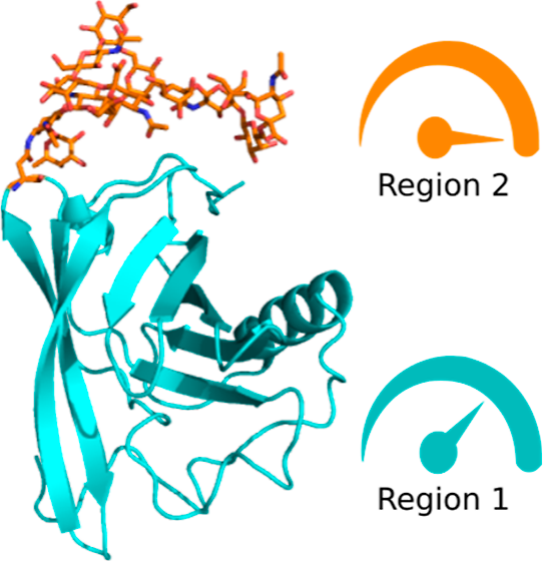

Molecular dynamics simulations often struggle to obtain sufficient sampling to study complex molecular events due to high energy barriers separating the minima of interest. Multiple enhanced sampling techniques have been developed and improved over the years to tackle this issue. Gaussian accelerated molecular dynamics (GaMD) is a recently developed enhanced sampling technique that works by adding a biasing potential, lifting the energy landscape up, and decreasing the height of its barriers. GaMD allows one to increase the sampling of events of interest without the need of a priori knowledge of the system or the relevant coordinates. All required acceleration parameters can be obtained from a previous search run. Upon its development, several improvements for the methodology have been proposed, among them selective GaMD in which the boosting potential is selectively applied to the region of interest. There are currently four selective GaMD methods that have shown promising results. However, all of these methods are constrained on the number, location, and scenarios in which this selective boosting potential can be applied to ligands, peptides, or protein–protein interactions. In this work, we showcase a GROMOS implementation of the GaMD methodology with a fully flexible selective GaMD approach that allows the user to define, in a straightforward way, multiple boosting potentials for as many regions as desired. We show and analyze the advantages of this flexible selective approach on two previously used test systems, the alanine dipeptide and the chignolin peptide, and extend these examples to study its applicability and potential to study conformational changes of glycans and glycosylated proteins.

## Introduction

Molecular dynamics (MD) simulations can contribute to the understanding of a wide range of biological phenomena thanks to their detailed spatial and time resolution.^1−4^ Specifically, it is of high relevance to be able to obtain free energies and free-energy landscapes of process of interest to better understand the mechanisms behind them.^5−10^ However, MD simulations often struggle to sufficiently sample the relevant conformational space of complex biomolecules. With the current computational resources that are commonly available, MD simulations are constrained to explore in a range of hundreds of nanoseconds to several microseconds. These time scales, although enough to study quick processes, are not enough to study a significant number of biologically relevant events, which happen in the milliseconds range due to the presence of high energy barriers.^3,11−13^

To tackle this limitation, several enhanced sampling methods have been developed that aim to increase the amount of sampling obtained without increasing the simulation time by adding a bias to the system.^14−22^ Enhanced sampling methods can be divided into two big categories, those that require a collective variable (CV) to operate and those that are CV independent. Although CV-based enhanced sampling methods provide higher sampling of the event of interest than the CV-independent ones, properly defining these CVs is not straightforward and often requires previous knowledge of the system, which may not be available in real-case scenarios.^23^

Gaussian-accelerated MD (GaMD) is a CV-independent method that has found diverse applications and aided the study of complex biological systems.^21,24−32^ GaMD works by adding a harmonic boosting potential to flatten the energy landscape and decrease the energy barriers.^21,33,34^ Because the boosting potential that is added follows a Gaussian distribution, GaMD simulations can be reweighted by using cumulant expansions to the second order, allowing one to unbias larger boosting potentials without running into statistical inaccuracies.^35,36^ Since its conception, several improvements of the GaMD methodology have been proposed and new variants of this method have been developed.^37−44^ Among the improvements on the methodology, a new subset of methods referred to as selective GaMD have shown an improved efficiency on the obtained sampling by selectively boosting a subset of the degrees of freedom of the system. Among these new methods, we find Pep-GaMD,^40^ in which a boosting potential is applied to a bound peptide and another one on the rest of the system, PPI-GaMD, in which the selective boosting potential is applied to the protein–protein interactions,^39^ LiGaMD, in which the selective booting potential is added to the ligand, and LiGaMD2, an improvement of the LIGaMD methodology, in which the selective boosting potential added to the ligand also includes the residues that are directly interacting with the ligand.^41,42^ Despite the improvement observed with the use of selective GaMD techniques, currently one is restricted to the existing selective boosting potentials that are available, which may not be useful for some scenarios in which the event of interest does not fall into any of these categories.

Here, we present a proof of concept of an expandable selective GAMD methodology that allows the user to flexibly select as many subsets of degrees of freedom as desired and define dedicated boosting potentials for them in a straightforward manner. Furthermore, we present the implementation of GaMD in the GROMOS software for biomolecular simulation.^45^ We also showcase some test cases and ideas of how selective GaMD can be used, the improvements in sampling that can be obtained, and an analysis of the mechanism involved in this increase in sampling. Examples include the acceleration of the solute and solvent independently or to study the highly dynamic nature of glycosylated proteins.

## Methodology

### Selective Gaussian Acceleration

The standard GaMD method works by adding a biasing potential, Δ*V*(*r*), to the potential energy, *V*(*r*) of the system, in order to flatten the energy landscape and decrease the energy barriers. The boosting potential follows a harmonic shape, as shown in eq 1, where *k* is the force constant and *E* is an energy threshold used to determine when the boosting potential is applied.^21^
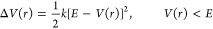
1

Considering a system with atoms at positions *r* and momenta *p*, the Hamiltonian of the system can be defined as in eq 2, where *K*(*p*) is the kinetic energy and *V*(*r*) is the potential energy. The potential energy is composed of bonded, nonbonded, and special terms.
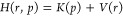
2

The bonded term is formed by the contribution of bonds, angles, and dihedral angles while the nonbonded term is composed of van der Waals and electrostatic interactions. These terms can be further split into the contributions of different sets of atoms. The nonbonded terms, nb, then become a combination of self-energy between the members of the same set, *V*_*i*–*i*_, and the interactions between two of the sets, *V*_*i*–*n*_, as shown in eq 3.
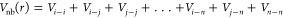
3

The various interactions (bonded *V*_*i*,b_, nonbonded self-energy *V*_*i*–*i*_, and nonbonded pair interactions *V*_*i*–*j*_) can be grouped together to form acceleration regions, that is, sets of potential energy terms for which a boosting potential will be defined and applied. By adding the boosting potential to these selected regions, one can achieve better sampling of the molecular events associated to them than using the standard GaMD approach, as seen in the previously published selective GaMD methods.^39−42^

In the methodology proposed in this work, the user has full flexibility to select the barriers to accelerate by first defining lists of atoms that should be considered as independent regions. The code automatically splits the energy terms as needed by monitoring *N* bonded terms, *N* nonbonded self-energy terms, and *N* (*N* – 1)/2 nonbonded interactions between groups, where *N* is the number of atom groups defined. Next, *M* acceleration regions are defined by indicating which terms should be treated together by using their indices, where *i*–*i* refers to the self-energy and bonded terms of group *i* and *i*–*j* refers to the nonbonded interaction between the groups with index *i* and index *j*. Examples on how to define these groups and regions can be found in the Supporting Information and a tutorial on how to use the proposed methodology can be found in the github version of the LiveCoMS tutorials for GROMOS.^46^ Once the M acceleration regions are defined, the user can select if they should be accelerated or not and which parameters (*E* and *k* in eq 1) to use for each of the biasing potentials, producing a set of *M* energy thresholds *E* and *M* force constants *k*. Additionally, the dihedral term of the bonded terms can be accelerated individually from the rest of the potential energy, which is usually referred as dual boosting.^21,33,34^ In case dual boosting is used, an extra set of *M* acceleration parameters is needed for the dihedral terms.

This setup allows to recreate any of the previously defined selective GaMD methods, while also allowing the user to more flexibly define selective acceleration potentials for the process of interest. For example, in the case of LiGaMD2,^41^ the same acceleration behavior could be obtained by defining two groups of atoms, one containing the ligand atoms and the atoms of the residues directly interacting with it and another group containing the rest of the system and then defining two boosting potentials: one for the group containing the ligand and another for the rest of the system. The parameters needed for each acceleration region can be obtained based on a search run by using the same approach described in the previous GaMD methodologies.^21,33,34^ Running a search simulation with the defined acceleration regions leads to suggestions for the choice of *E* (maximal observed energy for this region) and force constant *k* for each of the regions.

Thanks to the Gaussian distribution that the boosting potential follows, GaMD allows for unbiasing the simulations by reweighting using a cumulant expansion to the second order, circumventing the statistical noise that exponential reweighting methods tend to have due to a few high boosting energies dominating the denominator term.^35,36^ All the reweighting of the simulations in this work was performed using the pyreweight tools as previously described.^35^ The anharmonicity of the GaMD potential distribution was also assessed as a reliability measure for the reweighting.^21,33^ For the selected cases, the cumulant expansion reweighting was compared to an exponential averaging reweighting as implemented in gromos++,^47^ and no significant differences were observed.

### Studied Systems

**Figure 1:**
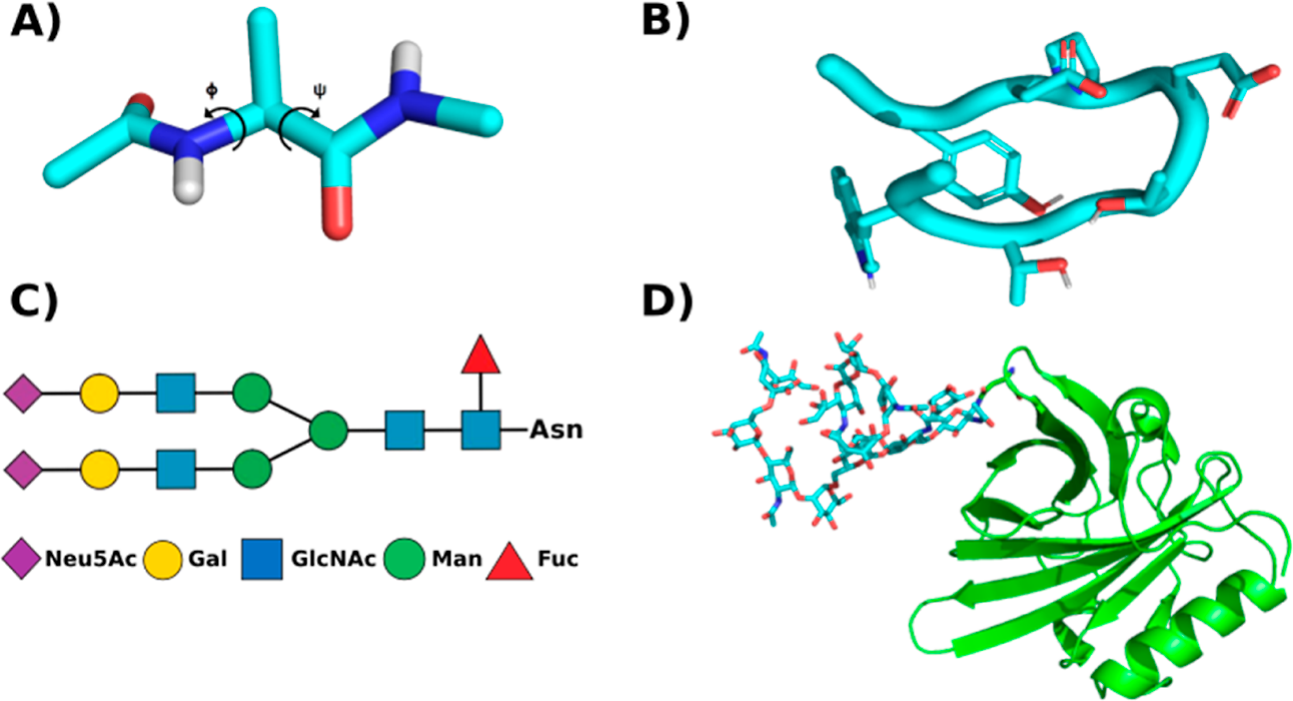
Systems under study. (A) Alanine dipeptide. (B) Folded structure of the chignolin peptide, 1UAO.^48^ (C) Schematic representation of the complex glycan NaNaF using the symbol nomenclature for glycans.^52^ (D) Olfactory panda protein, 5NGH,^53^ in green with the attached *N*-glycan NaNaF in cyan.

The alanine dipeptide as well as the chignolin peptide were used as validation systems for the described methodology because both systems have been extensively studied and used for the validation of the GaMD methodology.^21,33,34,48−50^ The alanine dipeptide (Figure 1a) allows for the construction of a detailed free energy landscape in accessible simulation times providing a good reference to assess the increase in sampling obtained by the use of other methodologies. The chignolin peptide is a short peptide of 10 amino acids (GYDPETGTWG) that folds forming a beta hairpin in a time scale accessible to MD (Figure 1b).^48,50,51^ The extended form of the chignolin peptide was used as starting coordinates. The NMR structure (1UAO) was used as a reference for the folded form of the peptide.^50^

The study of glycans is challenging due to the high conformational diversity that is thermodynamically accessible and the high energy barriers separating some of those conformations.^54^ To illustrate the capabilities of GaMD and selective GaMD on studying glycans, the conformations of the glycan tree NaNaF was used. NaNaF is a complex type glycan tree with additional *N*-acetyl-neuraminic acid groups and a 1,3-linked core fucose that is commonly found in mammals, as shown in Figure 1c. The NaNaF glycan was simulated in two different systems, NaNaF attached to the ASN residue of a short peptide (ANA) and NaNaF attached to residue Asn49 of a small globular mammalian protein, which is known to be glycosylated at that site (Figure 1d).^53^ The first system was used as a reference of all the conformations that could be seen while using different methods when there is no steric hampering or protein interactions, while the second system was used to illustrate how the selective GaMD could be used on a glycosylated protein of interest. The starting coordinates for the glycosylated protein were obtained from the crystal structure with pdb id 5NGH,^53^ the coordinates for the NaNaF glycosylation for both the peptide system and the protein were modeled as previously described.^54^

### Simulation Protocols

All the MD simulations were performed using the GROMOS simulation package^45^ (https://www.gromos.net) and the models were parametrized using the 54A8 GROMOS forcefield.^55^ The GaMD methodology as well as the described flexible selective GaMD approach were implemented in the GROMOS MD engine.

All systems were minimized using the steepest-descent algorithm in vacuum, and a periodic cubic water box of simple point charge water^56^ was added leaving 0.8 nm as buffer to the box edges. Counter ions were added by replacing water molecules with the most favorable electrostatic potential to reach charge neutralization using the programme ion provided in the gromos++ package.^47^ The systems were equilibrated with initial random velocities sampled from a Maxwell–Boltzmann distribution at 60 K and heated up to 300 K in five discrete steps. While heating up the system, position restraints on the solute atoms were reduced from 2.5 × 10^4^ to 0.0 kJ mol^–1^ nm^–2^. The systems were then further equilibrated at a constant pressure of 1 atm for 1 ns with an isothermal compressibility of 4.575 × 10^–4^ kJ^–1^ mol nm^3^. The different replicates were obtained by using different initial random velocities. All production runs were performed at a constant temperature and pressure of 300 K and 1 atm, respectively. Newton’s equations of motion were integrated using the leapfrog algorithm with a time step of 2 fs. The SHAKE algorithm^57^ was used to maintain the bond lengths at their optimal values. Long-range electrostatic interactions beyond a cut-off of 1.4 nm were truncated and approximated by a generalized reaction field^58^ with a relative dielectric permittivity of 61.^59^ Nonbonded interactions up to a distance of 0.8 nm were computed at every time step using a pair list that was updated every 10 fs. Interactions up to 1.4 nm were computed at pair list updates and kept constant in between. The GaMD acceleration parameters were obtained by performing a conventional MD (cMD) search of 1 ns in which statistics on the interaction energies were collected, followed by a GaMD search of 3 ns in which the acceleration parameters were periodically updated. GaMD production runs were performed with the acceleration parameters fixed to the values obtained from the search. All the acceleration parameters used can be found in the Supporting Information. For all GaMD runs, the dual boosting approach was used.^21^

### Local Elevation Simulations

Local elevation with umbrella sampling (US) potentials (LEUS) was used to obtain a reference of the thermodynamically accessible conformations of the glycan tree under study to compare to the cMD and GaMD simulations. Local elevation is a CV-dependent-enhanced sampling method, which defines the biasing potential with the sum of grid-based functions along the coordinates, *Q*, of the chosen CV, as shown in eq 4, where *g* is defined as a polynomial function, as shown in eq 5.^17,60^
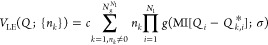
4
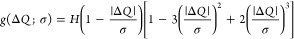
5*N*_g_ is the number of grid points along the *N*_l_ dimensional subspace and *n*_*k*_ is the number of visits to that corresponding grid cell. MI is the minimum image function to account for periodicity of *Q* and Q* is the value of *Q* on a grid point. *H* is the Heaviside step function. The force constant, *c*, and width, σ, are the free parameters that need to be tuned for the building-up procedure. In the LEUS methodology, the LE phase is followed by an equilibrium US phase, during which the values of the number of visits are no longer increased making the biased Hamiltonian time independent for this phase.

For this study, the chosen CVs that define the LEUS subspace were the dihedral angles ϕ and ψ of the glycosidic linkages, with the following LEUS parameters *N*_g_ = 36, σ = 360°/*N*_g_, *c* = 0.005 kJ mol^–1^, and *N*_l_ = 2. The LE phases were run for 100 ns for all relevant disaccharides in our previous work.^54^ The LE biases were subsequently used in the US phase for the entire glycan with linkage-specific biases.

### Glycosylation Conformational Analysis

To classify the conformations of the glycan tree observed during the simulations, time series of the φ, ψ, and ω dihedral angles were calculated for all the glycosidic linkages. Since each linkage has a specific free-energy landscape associated to those dihedrals, the conformations observed in each configuration can be encoded by a string of letters (A, B, C, or D) assigned based on the minima that each of the sets of dihedrals fall into.^54,61,62^ This classification has been updated for 1 → 6 linkages and the effect of periodicity was taken into account. This addition allows one to properly account for the extra degrees of freedom that some of the glycosidic linkages have, such as the d-Man-α-(1 → 6)-d-Man, d-Neu5Ac-α-(2 → 6)-d-Gal, and d-GlcNAc-β-(1 → N).^63^ A more detailed description of the classification scheme used along with the dihedral angle definitions and free energy profiles can be found elsewhere.^63^

The string encoding for the glycosidic linkages allows one to easily cluster the conformations of the glycans during the MD simulation and to assess how diverse and complete the sampling was by using the Hamming distance between them. For this study, the number of different clusters was monitored over time together with the number of transitions between clusters. The LEUS simulations were used as an upper threshold of the accessible diversity of the glycan conformations.

## Results

### Alanine Dipeptide

**Figure 2:**
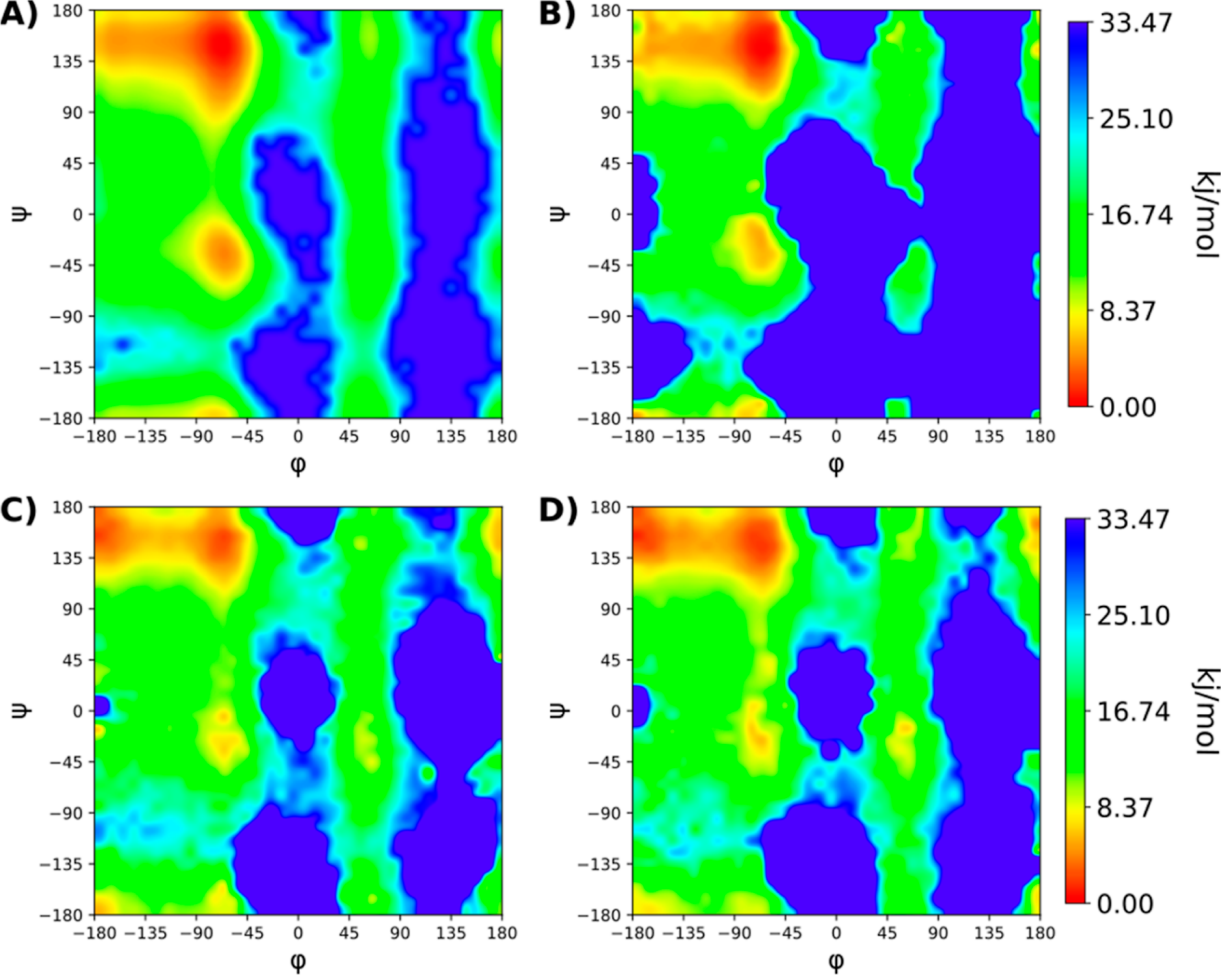
(A) Free-energy landscape over the φ and ψ dihedrals for the 5 μs of aggregated cMD data. (B) Free-energy landscape for the 500 ns of aggregated reweighted dual boosting GaMD simulations. (C) Free-energy landscape for the 500 ns of aggregated reweighted selective GaMD simulations in which a boosting potential was applied selectively to the potential energy terms involving alanine dipeptide atoms and an additional potential was applied to the water–water interactions. (D,C) Free-energy landscape for the 500 ns of aggregated reweighted selective GaMD simulations in which the selective boosting potential was applied only to the alanine dipeptide.

The free energy landscape of the alanine dipeptide over its φ and ψ dihedral angles was computed and compared using four different approaches. Five replicates of conventional MD simulations of 1 μs each were used to obtain a converged reference free-energy landscape of the alanine dipeptide, Figure S1 in the Supporting Information. Five replicates of 100 ns each of standard dual-boost GaMD and two sets of five replicates of 100 ns each of selective GaMD were performed. In the selective GaMD runs, two acceleration regions were defined, one containing all the interactions involving the alanine dipeptide and a second one with the water–water interactions. On the first selective GaMD set both regions were accelerated independently, while in the second set only the acceleration group containing the alanine dipeptide was accelerated. The free energy landscape of the standard GaMD still lacked some of the regions that were explored during the long cMD simulations, especially in the less probable areas of the ψ dihedral at 45°. On the other hand, both selective GaMD setups achieved better coverage of the energy landscape reproducing the energy landscape obtained with the long cMD simulations, as shown in Figure 2. The discrepancies between the long cMD run and the selective GaMD centered mostly around the edges of the less frequent conformations, confirming that the selective approaches correctly recovered the original energy landscape, as shown in Figure S2.

The standard GaMD approach produced an average boosting potential of 36.6 ± 8.5 kJ/mol, while the boosting applied on the alanine dipeptide for both selective GaMD runs was slightly higher with a boosting potential of 44.8 ± 9.2 kJ/mol for both runs. The anharmonicity of the boosting potential distribution was low for all cases, 8.6 × 10^–3^ for the standard GaMD approach, 6.1 × 10^–3^ for the selective GaMD setup in which both the alanine dipeptide and the water–water interactions were accelerated, and 6.2 × 10^–3^ for the selective GaMD setup with only the alanine dipeptide being accelerated. The narrow boosting potential distribution together with the low anharmonicity guaranties an accurate reweighting.^21^ A similar performance obtained with both GaMD setups hints, that for this system, the energy barriers come principally from the dipeptide itself and not from water rearrangements.

### Chignolin Peptide

The free energy landscape of the folding event for the chignolin peptide can be described using the RMSD to the folded structure and radius of gyration as CVs for the free energy landscape.^21,33^ Similar to the alanine dipeptide, four setups were tested, cMD, standard GaMD, and two selective GaMD with five replicates of 100 ns each. Similar to the alanine dipeptide, the two selective GaMD sets had two acceleration regions defined, one containing all the interactions involving the chignolin peptide and one with the water–water interactions. The first set boosted both acceleration regions while the second only boosted the group containing the chignolin peptide.

**Figure 3:**
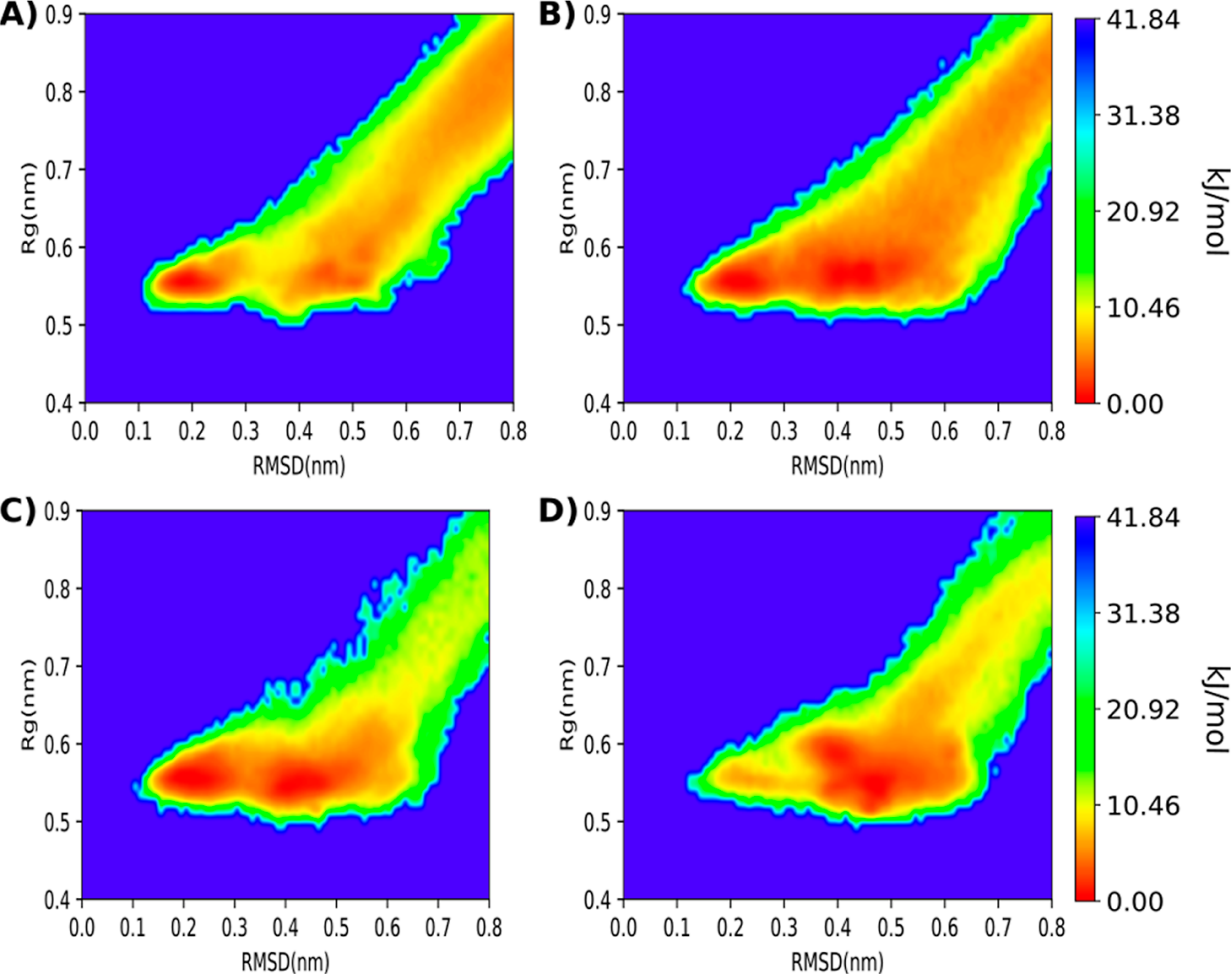
(A) Free-energy landscape of the folding event of the chignolin peptide for the 500 ns of aggregated cMD simulations. On the *X* axis the RMSD of the chignolin peptide to the reference NMR structure^50^ and on the *Y* axis the radius of gyration nm. The obtained free-energy landscape lacks a clear definition of the two expected energy minima which cannot be differentiated from the rest. (B) Free-energy landscape of the folding event for the 500 ns of aggregated reweighted GaMD simulation. (C) Free-energy landscape for the 500 ns of aggregated reweighted selective GaMD simulation in which one boosting potential was applied selectively to the potential energy terms involving atoms of the chignolin peptide and another boosting potential to the solvent–solvent interactions. The free energy landscape shows the two expected energy minima corresponding to the completely folded structure at RMSD 0.1–0.2 nm and the intermediate folded structure at around RMSD 0.4 nm.^21,33,48,50^ (D) Free-energy landscape for the 500 ns of aggregated reweighted selective GaMD simulation in which the boosting potential was applied selectively to the potential energy terms involving atoms of the Chignolin peptide.

The free-energy landscape as well as the folding events observed were tracked, see Figure 3. The two energy minima corresponding to the intermediate state and folded state were used to monitor the folding events.^21,33,48,50^ A folding event was defined as the occurrence of conformations within 0.3 nm RMSD from the NMR structure, with a lifetime of at least 100 ps. For the cMD simulations, three of the five replicates managed to reach the folded state (one folding event each), while the other two remained unfolded. Accordingly, the free energy landscape obtained did not show significant minima other than the folded state. Four of the GaMD simulations managed to reach the folded state and unfolded shortly after. The free energy landscape obtained was also missing the expected minima. All the selective GaMD simulations, in which both the peptide and the solvent were accelerated separately, reached the folded state. In addition to the folding events, these simulations also showcased unfolding events to the intermediate state and folded back again from the intermediate state to the folded state. The obtained free energy landscape showed the two expected energy minima better defined. The selective GaMD simulations in which the boosting potential was applied only to the interactions involving the chignolin peptide showcased only two short lived folding events and remained for most of the simulation time in the partially folded state, as shown in Figures 3 and S3.

**Figure 4:**
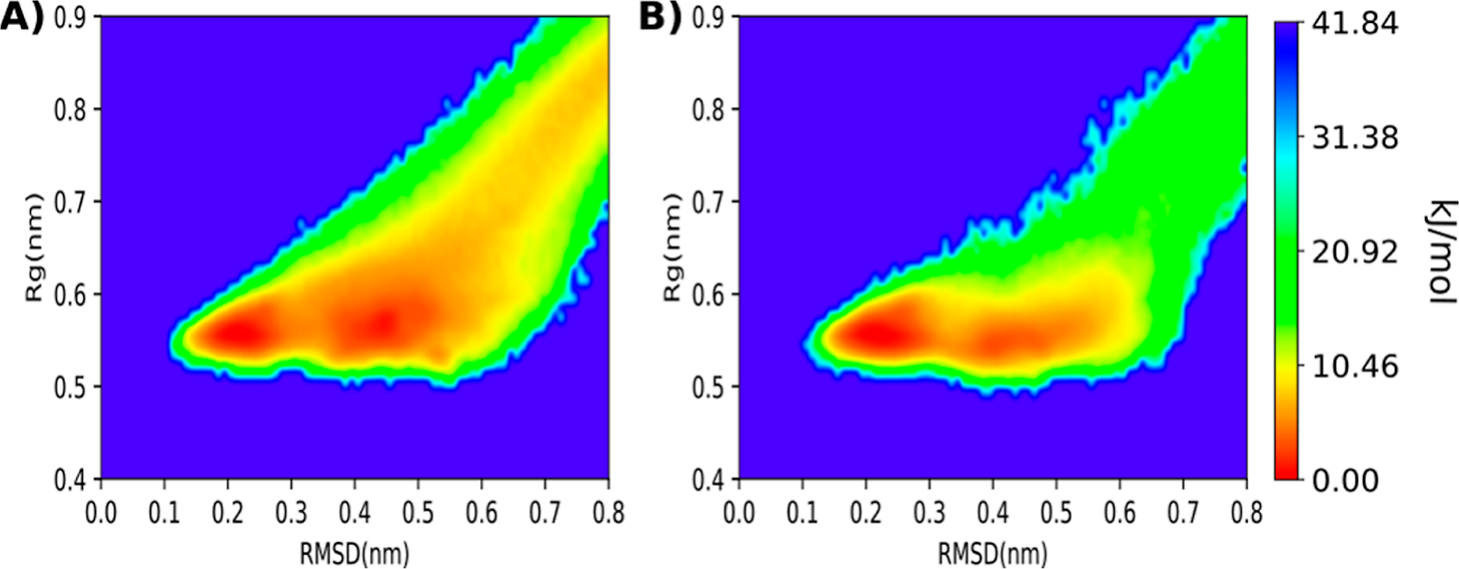
Free-energy landscape of the folding event of the chignolin peptide for 1.5 μs of aggregated reweighted GaMD simulation, panel A, and 1.5 μs of selective GaMD, panel B. On the *X* axis, the RMSD of the chignolin peptide to the reference NMR structure^50^ and on the *Y* axis the radius of gyration. Both free-energy landscapes show the two expected minima corresponding to the completely folded structure at RMSD 0.1–0.2 nm and the intermediate folded structure at around RMSD 0.4 nm.^21,33,48,50^

The two sets of simulations that performed the best (GaMD and selective GaMD with two acceleration regions) were extended to 300 ns to achieve better convergence, as shown in Figure S4. Both setups yielded a similar free energy landscape with the two expected minima well defined, as shown in Figure 4. The selective GaMD simulations displayed more folding and unfolding events than the GaMD using the standard approach. The GaMD approach led to a more pronounced tail in the free-energy landscape at more extended conformations. This is due to the slower initial folding events, leading to stronger contributions from the initial (extended) structure. In total, 142 folding events were observed in the selective GaMD runs and 77 in the standard GaMD simulations.

The average boosting potential for the standard GaMD simulations was 19.2 ± 7.5 kJ/mol. Both selective GaMD sets had a similar boosting potential applied to the interactions involving the peptide (19.7 ± 7.9 kJ/mol for the simulations in which both the chignolin and the solvent were selectively accelerated and 18.2 ± 7.5 kJ/mol for the selective GaMD runs in which only the interactions involving the chignolin peptide were accelerated). The anharmonicity of the boosting potential distributions were low (anharmonicity < 2.9 × 10^–2^) for all the sets of simulations allowing for an accurate reweighting.^21^

### NaNaF Glycosylation

Five replicates of 100 ns of conventional MD, standard GaMD, and two sets of selective GaMD of the NaNaF glycan were performed. The first selective GaMD set had two selective boosting potentials, one applied to the interactions involving the glycan tree and another accelerating the solvent–solvent interactions. The second set of selective GaMD simulations only had one potential applied to the interactions involving the glycan tree. An additional run of 100 ns of LEUS was performed as a reference to have an upper threshold of the conformations accessible.

**Figure 5:**
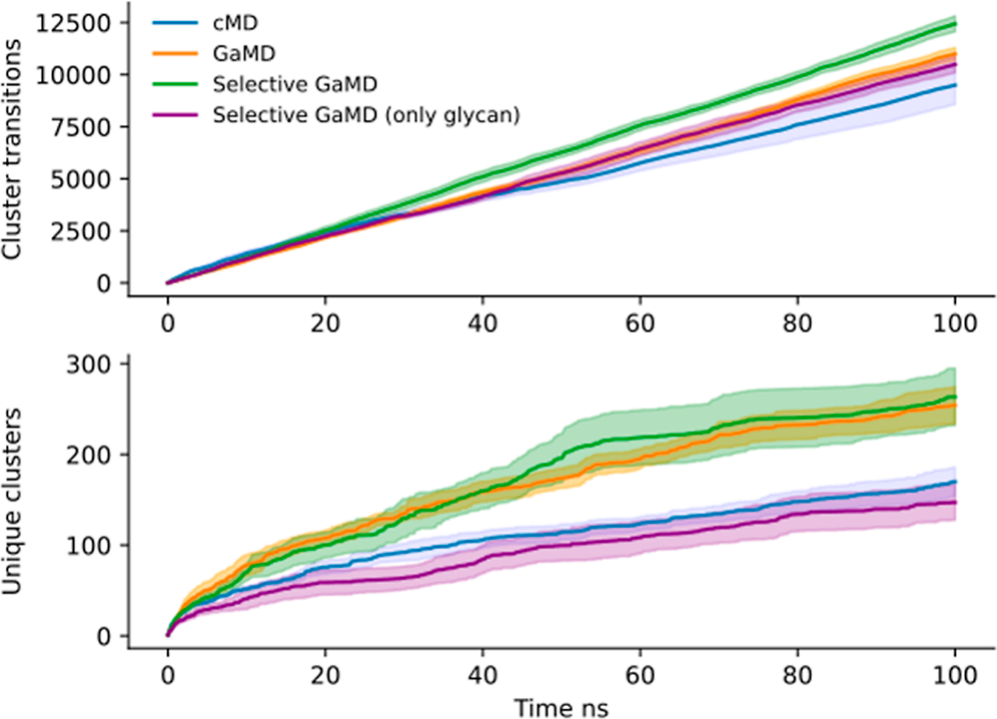
Average number of transitions between conformation clusters (top panel) and average number of unique conformation clusters (bottom panel) of the glycan, observed over the simulation length. In blue, the results for the cMD simulations. In orange, the results for the GaMD simulations using the standard approach. In green, the selective GaMD runs in which two acceleration regions were defined, one containing all the interaction involving the glycan and another with the solvent–solvent interactions. In purple, the selective GaMD simulations in which the selective boosting was applied only to the interactions involving the glycan. The shaded area indicates the respective standard error of the mean.

The φ, ψ, and ω dihedral angles of the glycan tree were calculated and the trajectories were encoded into letters based on those dihedrals. These encoded letter strings represent the different conformations sampled by the glycan as previously described.^54,62,63^ The number of unique conformations and number of transitions between conformations was monitored over time (Figure 5). The LEUS simulation sampled 13,139 different conformations. However, a LEUS simulation samples a significant number of conformations that are rarely accessible. To filter out these unlikely unfavorable conformations, a better metric would be to see how many of those conformations have been sampled for at least 10 ps. In total, from those 13,139 conformations, 1063 fulfilled this sampling criteria, providing a better picture of the number of conformations that one may expect to see. The cMD runs sampled a total of 477 unique conformations and shifted between conformations at a rate of 95 transitions per ns totaling 9496 transitions per simulation with an average of 170 different conformations seen per simulation. The standard GaMD simulations sampled significantly more conformations than the cMD with a total of 606 unique clusters observed (*p* < 0.02). The number of transitions observed was 10,994 per simulation, with an average of 110 transitions per ns and an average of 254 different conformations seen per simulation. The first set of selective GaMD runs showcased a similar sampling as the standard GaMD runs, 680 conformations, with an average of 263 different conformations seen per simulation, but displayed significantly more transitions between clusters (*p* < 0.03) with 12,435 observed transitions per simulation and an average of 124 transitions per ns. Finally, the selective GaMD, in which the selective boosting potential was applied only to the glycan tree, sampled less conformations than the other GaMD simulations, with 372 unique clusters found, and showcased a similar number of transitions to the standard GaMD simulations, with an average of 105 transitions per ns, a total of 10,491 transitions, and average of 147 different conformations seen per simulation (Figure 5).

The average boosting potential for the standard GaMD run was 18.5 ± 7.3 kJ/mol. Both sets of selective GaMD runs received, a similar boosting potential to the glycan tree with 20.3 ± 7.7 kJ/mol for the selective GaMD with two acceleration regions and 19.9 ± 7.6 for the selective GaMD set in which only the glycan tree was accelerated. Interestingly, the selective GaMD with two separate accelerations for the glycan and the solvent leads to the highest number of transitions, while the selective approach in which only the glycan is accelerated is comparable to regular GaMD. This suggests that acceleration of the solvent–solvent interactions is relevant for the glycan dynamics, e.g., due to the viscosity of the solvent, or the need to readjust the solvation shells in different glycan conformations. A separate acceleration avoids compensating effects between the two regions that may occur in regular GaMD and still leads to a speed-up.

### *N*-Glycosylated Protein

Four sets of five 100 ns simulations were performed on the *N*-glycosylated protein 5NGH.^53^ In this case, the tested setups were cMD, GaMD, and two different sets of selective GaMD simulations. On the first selective GaMD set, the system was split into two acceleration regions, one containing the interactions involving the *N*-glycosylated protein and another one containing the solvent–solvent interactions. In the second set of selective GaMD simulations, the solute was further split in two acceleration regions, one containing all the interactions involving the atoms of the glycan tree and another one containing the protein–protein and protein–solvent interactions. This setup resulted in three independent sets of boosting potentials.

**Figure 6:**
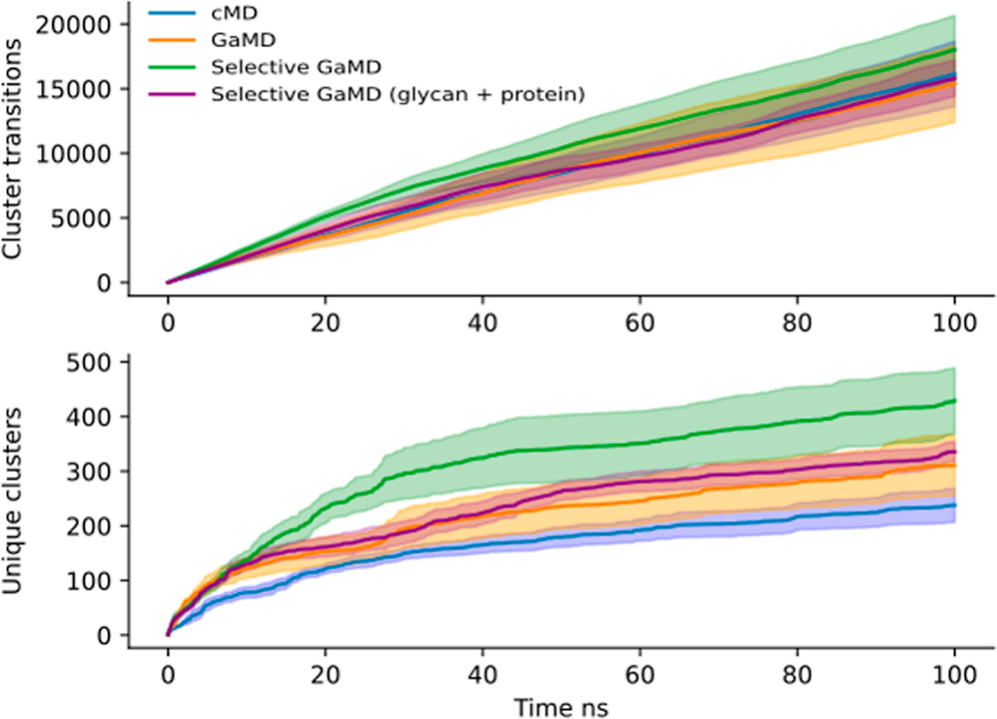
Average number of transitions between conformation clusters (top panel) and average number of unique conformation clusters (bottom panel) of the glycan, observed over the simulation length. In blue, the results for the cMD simulations. In orange, the results for the GaMD simulations using the standard approach. In green, the selective GaMD runs in which two acceleration regions were defined, one containing all the interaction involving the solute and another with the solvent–solvent interactions. In purple, the selective GaMD simulations in which three acceleration regions were defined, one with the interactions involving the glycan tree, another one containing the protein–protein and protein–solvent interactions, and a final one containing the solvent–solvent interactions. The shaded area indicates the respective standard error of the mean.

All the tested setups produced a similar number of cluster transitions with an average of 16,336 transition per simulation, as seen in Figure 6. All the GaMD approaches tested, with the exception of the standard GaMD, sampled significantly more clusters than the cMD simulations (*p* < 0.05). With 929 unique clusters found in the cMD simulations (237 in average per simulation), 1330 for the standard GaMD ones (310 in average per simulation), 1795 for the selective GaMD with two acceleration regions, and 1374 for the selective GaMD setup with three acceleration regions (429 and 335 in average per simulation, respectively).

All the GaMD approaches sampled a similar number of unique clusters with no significant differences observed (*p* > 0.2). The standard GaMD setup showed one outlier simulation that got trapped in a local minimum and only sampled 89 different conformations over the 100 ns of simulation, 277 less clusters than the average number of clusters found in the other replicates, 366. The selective GaMD with three acceleration regions produced more consistent results across simulations. When correcting for the observed outlier, all the GaMD approaches sampled significantly more unique clusters than the cMD simulations (*p* < 0.05).

**Figure 7:**
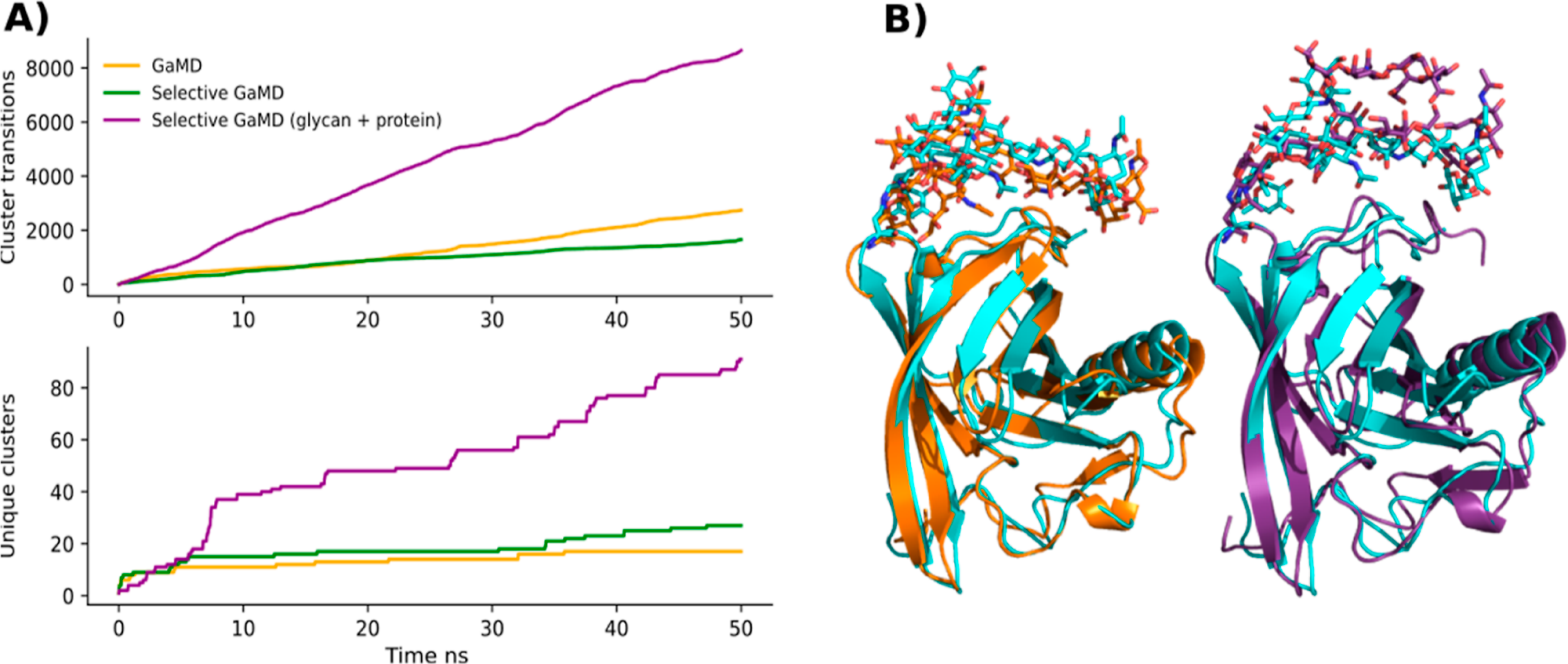
(A) Average number of transitions between conformation clusters (top panel) and average number of unique conformation clusters (bottom panel) observed over the simulation length of the simulations starting from the conformation that was stuck in a deep local potential-energy minimum. In orange, the results for the GaMD simulations using the standard approach. In green, the selective GaMD runs in which two acceleration regions were defined, one containing all the interaction involving the solute and another with the solvent–solvent interactions. In purple, the selective GaMD simulations in which three acceleration regions were defined, one with the interactions involving the glycan tree, another one containing the protein–protein and protein–solvent interactions, and a final one containing the solvent–solvent interactions. (B) Initial conformation of the simulations in A in cyan, final snapshot of the GaMD simulations in orange, and final snapshot for the selective GaMD with which three acceleration regions were defined in purple.

To test the capabilities of the different GaMD approaches to escape possible deep minima, the snapshot after 50 ns of the standard GaMD simulation that seemed to get trapped in a single conformation was used as the starting point to test other GaMD setups under study. The standard GaMD simulation was not able to escape this conformation in the subsequent 50 ns of simulation. Only the selective GaMD setup in which the glycan tree was accelerated separately from the rest of the system manages to escape the deep minima. This simulation had three times more transitions between clusters and sampled more than three times more conformations than the other GaMD approaches, as seen in Figure 7.

The average boosting potential for the standard GaMD run was 15.3 ± 6.9 kJ/mol. Both sets of selective GaMD runs received a similar boosting potential to the glycan tree with 18.1 ± 7.4 kJ/mol for the selective GaMD with two acceleration regions and 14.9 ± 6.7 for the selective GaMD set with an extra acceleration region for the glycan tree.

## Discussion

The selective GaMD algorithm works by adding a boosting potential to the chosen interaction terms of the potential energy. By doing so, it is possible to achieve a higher sampling of the events of interest than the sampling produced by the standard GaMD approach. The current selective GaMD methodologies are restricted to which regions of the system they can be applied to, limiting the number of scenarios in which this methodology can be successfully applied.^39−42^ In addition to making the methodology applicable to other biological systems, being able to flexibly define the degrees of freedom to accelerate allows for better performance as seen in the LiGaMD2 algorithm whose main improvement when compared to the original LiGaMD methodology is the inclusion of the protein residues in the binding pocket to the selective boosting potential used on the ligand.^41^

In this work, we present a prototype of a flexible selective GaMD approach and validate it with the alanine dipeptide, chignolin peptide, the glycan NaNaF, and a *N*-glycosylated protein.^48,49,53^ The proposed methodology offers a user-friendly way to design unconstrained acceleration regions for both the atoms to accelerate and the number of boosting potentials to use, while also offering full control on which interaction terms to accelerate.

For all the systems under study, using two boosting potentials, one applied to the solute and another to the solvent, produced the best results, suggesting that this approach could be applied to any system of interest with ease to obtain higher enhancing than with the standard GaMD method. The performed simulations in which the solvent–solvent interactions were not accelerated underperformed for some of the systems under study, especially when conformational changes were of relevance. For example, in the case of the chignolin peptide, the peptide reached the partially folded state but struggled to reach the complete folded state, suggesting that for the last step of the folding event the energetic barriers of the solvent–solvent interactions are of high importance. Similarly, the number of conformations observed for the glycan NaNaF without accelerating the solvent–solvent interactions was comparable to conventional MD.

The current work shows that splitting the system in additional acceleration regions not only allows for an increased acceleration but also helps focusing the acceleration potential to barriers of interest. When an acceleration region contains a large number of atoms, the resulting potential energy term is noisier, and the contribution to the energy term of the degrees of freedom of interest gets masked by the other energy terms. This results in a boosting potential that is less sensible to the energy landscape of the region of interest, which leads to less acceleration for possibly deep energy minima than desired. The outcome is that systems may remain trapped sampling a small conformational space as seen in the test case of the *N*-glycosylated protein. For the *N*-glycosylated protein, one of the simulations reached a local minimum. Once this local minimum was reached, both the standard GaMD approach and the selective GaMD approach with two acceleration regions (one for the solvent and another for the solute) remained trapped constantly sampling the same conformations of the glycan tree. The boosting potential applied on these approaches showed no correlation with the energies involving the glycan tree, with *R*^2^ = 0.009 and 0.03, respectively. This suggests that a strong favorable glycan–protein interaction determined the sampling, while the acceleration was applied to the joint glycan and protein degrees of freedom. On the other hand, the system that had an additional acceleration region containing only the interactions involving the glycan tree managed to escape the local minima, and the boosting potential perfectly correlated (*R*^2^ = 1.0) with the energy landscape of the glycan tree at low potential energy values.

Having this extra sensibility to detect barriers in the energy landscape, makes the selective GaMD approach appealing to use for systems in which the overall degrees of freedom or region of interest are known but there is not enough previous knowledge on the cause of those energy barriers to properly define distinct CVs. Note that in the limit of defining an acceleration region that consists of a single degree of freedom, GaMD becomes a CV-dependent enhanced sampling method.

Aside from the naïve use case of separating solute and solvent into two acceleration regions and the already described selective GaMD uses,^39−42^ the suggested flexible approach could be used, for example, to aid in the study of glycosylation, membrane proteins, and proteins with unordered regions among others, by separating the glycan, the membrane, or the unordered regions from the rest of the protein. In addition, the proposed flexible selective GaMD approach could be used to recreate any of the previous selective GaMD methods by defining the same regions, while also allowing more control on how the interactions between the regions will be treated.

It is important to notice that enhancing the system could lead to undesired events such as misfolding occurring, and the simulations using selective GaMD need to be examined carefully before extracting conclusions from them. By accelerating only selected sets of degrees of freedom, the risk of unintentionally unfolding a protein will be significantly reduced.

In summary, the proposed flexible selective GaMD approach allows for a straightforward way to define multiple acceleration potentials for the regions of interest, circumventing the original restrictions of the selective GaMD approach and making it applicable to a broader range of systems. In this work, we showcased its potential use to study glycosylation, but the extra sensibility that the selective GaMD offers can be used to any system of interest, only requiring the list of atoms that one wants to selectively accelerate, e.g., a loop or the flexible termini of a protein. In addition, this methodology could be further expanded by combining it with other reported improvements of the GaMD methodology such as replica exchange GaMD^37^ or the use of sigmoid acceleration.^64^

## Supporting Information Available

Acceleration parameters, examples on how to define the acceleration groups and regions, error estimates for all the PMFs present, and discrepancies between the long cMD alanine dipeptide run and the different tested setups (PDF)
GaMD files (ZIP)

## Notes

All topological information, initial structures, input files, and equilibrated coordinates are made available in the Supporting Information. All simulations were performed with the GROMOS suite of programs for molecular simulations, which is available free of charge at www.gromos.net.
