# Supplementary material for: Flexible Gaussian Accelerated Molecular Dynamics to Enhance Biological Sampling
Source: J Chem Theory Comput. 2023 Aug 31;19(18):6521–31. doi: 10.1021/acs.jctc.3c00619 (PMC10536968; doi:10.1021/acs.jctc.3c00619)
Supplement: Supplementary file 1 — ct3c00619_si_001.pdf [file ct3c00619_si_001.pdf]

Supporting Information: Flexible Gaussian accelerated molecular dynamics to enhance biological sampling

Oriol Gracia Carmona,[a] Chris Oostenbrink[a,b]*

a. *Institute for Molecular Modeling and Simulation, Department of Material Sciences and Process Engineering, University of Natural Resources and Life Sciences, Vienna. Muthgasse 18, 1190 Vienna Austria*
b. *Christian Doppler Laboratory for Molecular Informatics in the Biosciences, University of Natural Resources and Life Sciences, Vienna. Muthgasse 18, 1190 Vienna, Austria*

** Corresponding author: chris.oostenbrink@boku.ac.at*

Alanine dipeptide error estimation

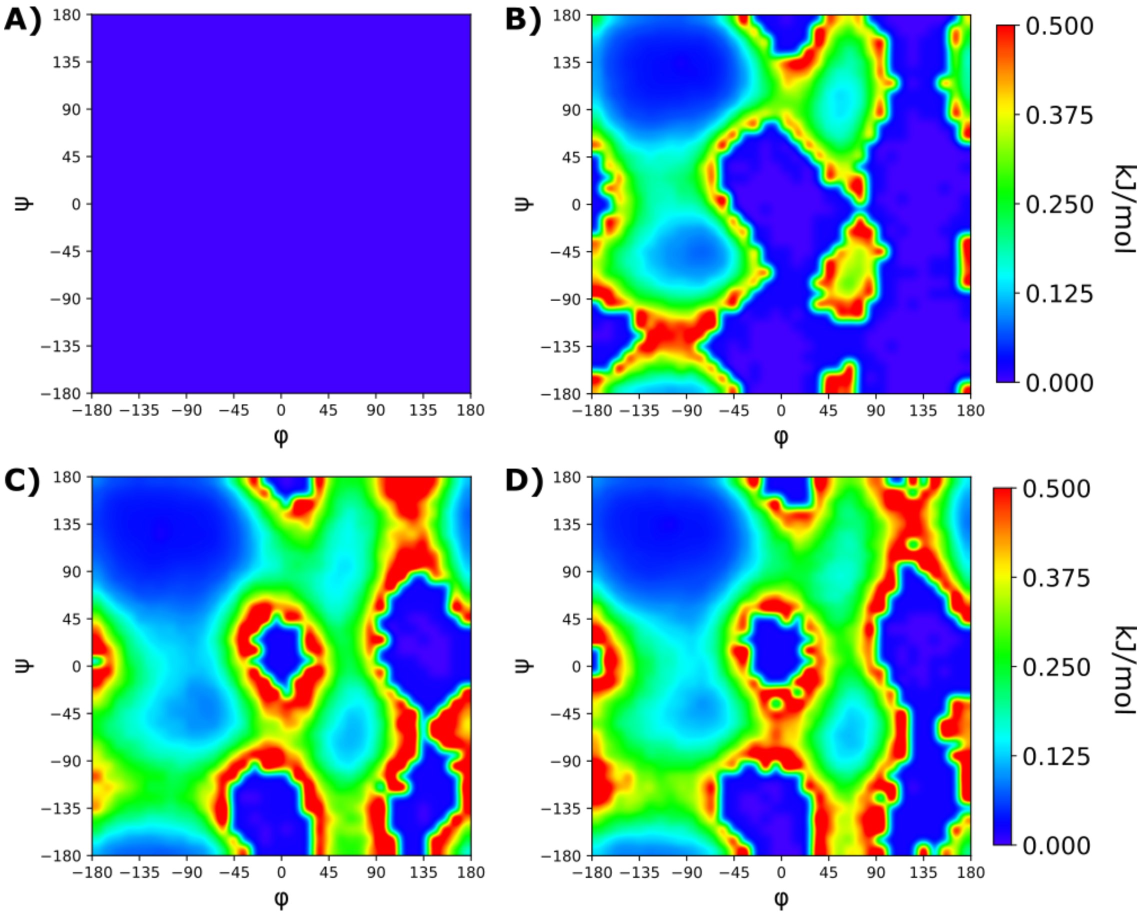

Figure S1. Standard deviation of the alanine dipeptide PMF's for the cMD runs (A), the standard GaMD run (B), the selective GaMD runs in which the solute and solvent were separated into two acceleration groups (C) and the selective GaMD in which only the solute was accelerated (D). The standard deviations were estimated using 1000 bootstrap samples.

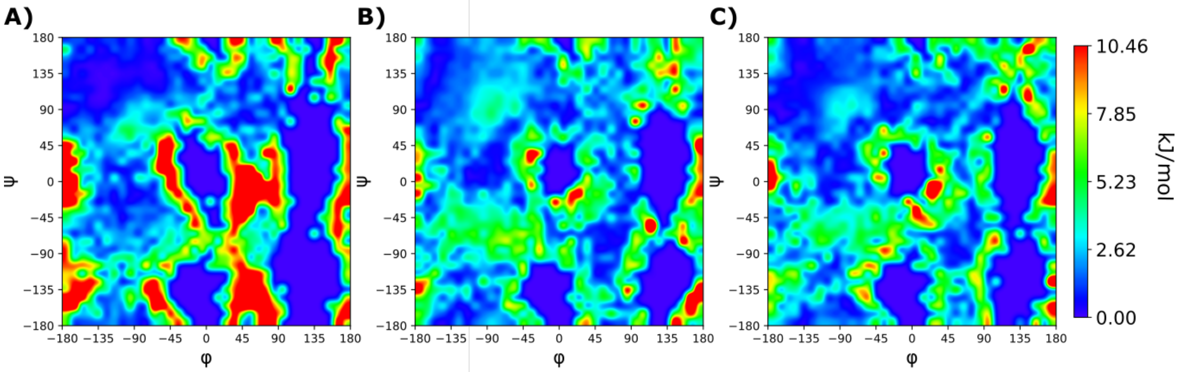

Figure S2. Free energy difference between the 5µs cMD PMF and the PMF's of the standard GaMD run (A), the selective GaMD runs in which the solute and solvent were separated into two acceleration groups (B) and the selective GaMD in which only the solute was accelerated (C).

Chignolin error estimation

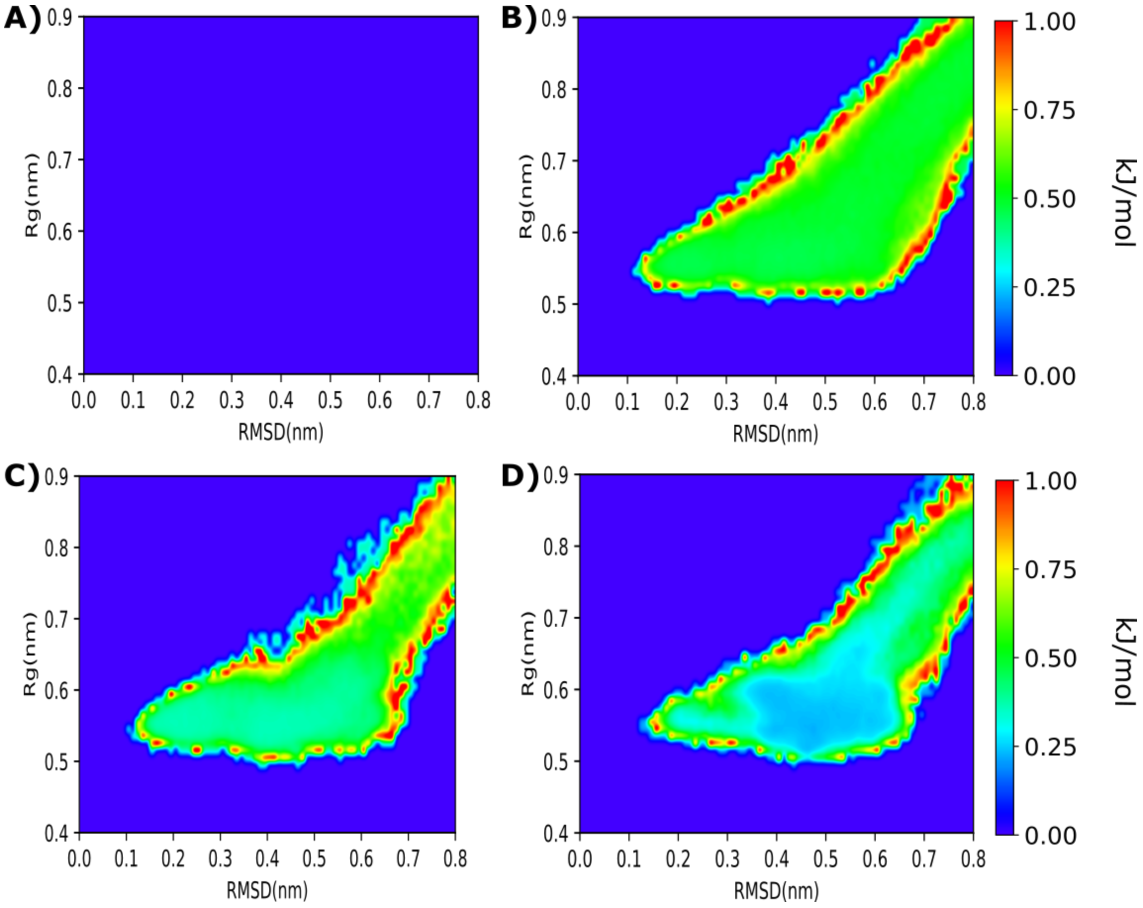

Figure S3. Standard deviation of the chignolin peptide PMF's for the cMD runs (A), the standard GaMD runs (B), the selective GaMD runs in which the solute and solvent were separated into two acceleration groups (C) and the selective GaMD in which only the solute was accelerated (D). The standard deviations were estimated using 1000 bootstrap samples.

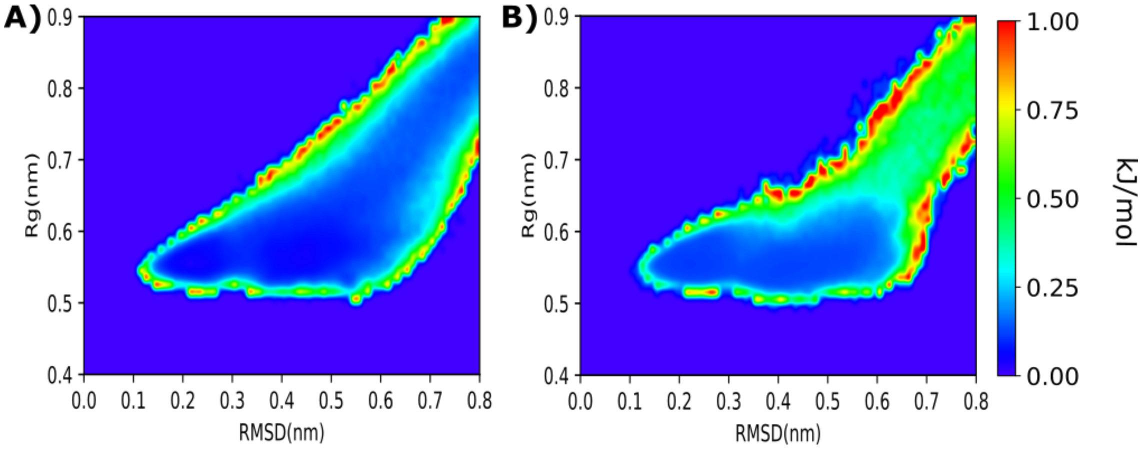

Figure S4. Standard deviation of the elongated chignolin peptide PMF's obtained from 1.5µs of aggregated standard GaMD runs (A) and from 1.5µs of aggregated selective GaMD runs in which the solute and solvent were separated into two acceleration groups. The standard deviations were estimated using 1000 bootstrap samples.

Acceleration regions and parameters

The required input files to define the acceleration groups and regions have the following structure. In the first block, GAMDATOMS, the number of groups of atoms to account for separately are specified by using their indexes ranges, resulting in three values, (INATOM) which indicates the index of the first atom belonging to that acceleration group, (FINATOM) which indicates the index of the last atom of the group and (AGROUP) which is the integer for that acceleration group. In the case of standard GaMD, only one set of atoms is defined while for selective GaMD, the user can define as many atom groups as desired.

The next block, GAMDGROUPS, specifies the number of distinct acceleration potentials to use. This is done by indicating the integers that identify each of the acceleration groups previously defined. The pairs of groups whose interactions should be accelerated together are specified using their integers, (AGROUP_1, AGROUP_2) with an additional integer to specify to which acceleration region those interactions should be assigned, ACCELGROUP. For example, AGROUP_1 = 1, AGROUP_2 = 2 and ACCELGROUP = 1 will assign the interactions of the groups 1 and 2 to the first boosting potential. A more detailed tutorial on how to use the presented methodology can be found in the github version of the LiveCoMS tutorials for GROMOS[1].

Alanine dipeptide

Acceleration groups, acceleration regions and parameters used for the different setups of the Alanine dipeptide simulations

Standard GaMD:

```
TITLE
GAMD_all_atoms
END
GAMDATOMS
1
#   INATOM    FINATOM    AGROUP
        1       3840         1
END
GAMDGROUPS
1
# GROUP_1    GROUP_2    ACCELGROUP
        1          1             1
END
```

Energy threshold and force constant for the potential energy term: -49549.22 kJ/mol, 2.56333526 * 10^{-5} mol/kJ.
Energy threshold and force constant for the dihedral term: 124.52 kJ/mol, 8.520282 * 10^{-3} mol/kJ.

Selective GaMD (two acceleration regions, solute and solvent):

```
TITLE
```

```
GAMD by parts
END
GAMDATOMS
2
#   INATOM   FINATOM   AGROUP
        1        12        1
       13      3840        2
END
GAMDGROUPS
2
# GROUP_1   GROUP_2   ACCELGROUP
        1         1            1
        1         2            1
        2         2            2
END
```

Energy threshold and force constant for the potential energy term of the first acceleration region: 327.72 kJ/mol, 1.188933 * 10^{-3} mol/kJ.
Energy threshold and force constant for the dihedral term of the first acceleration region: 147.22 kJ/mol, 7.11499 * 10^{-3} mol/kJ.
Energy threshold and force constant for the potential energy term for the second acceleration region: -50592.9 kJ/mol, 4.85777 * 10^{-5} mol/kJ.

Selective GaMD (one acceleration region for the alanine dipeptide only):

```
TITLE
GAMD by parts
END
GAMDATOMS
2
#   INATOM   FINATOM   AGROUP
        1        12        1
       13      3840        2
END
GAMDGROUPS
1
# GROUP_1   GROUP_2   ACCELGROUP
        1         1            1
        1         2            1
END
```

Energy threshold and force constant for the potential energy term: 327.72 kJ/mol, 1.188933 * 10^{-3} mol/kJ.
Energy threshold and force constant for the dihedral term: 147.22 kJ/mol, 7.11499 * 10^{-3} mol/kJ.

Chignolin

Acceleration groups, acceleration regions and parameters used for the different setups of the Chignolin simulations.

Standard GaMD:

```
TITLE
GAMD_all_atoms
END
GAMDATOMS
1
#   INATOM   FINATOM   AGROUP
         1     22624        1
END
GAMDGROUPS
1
# GROUP_1   GROUP_2   ACCELGROUP
        1         1            1
END
```

Energy threshold and force constant for the potential energy term: -310580.729 kJ/mol, 1.94500018 * 10^{-5} mol/kJ.
Energy threshold and force constant for the dihedral term: 308.026578 kJ/mol, 6.61061472 * 10^{-3} mol/kJ.

Selective GaMD (two acceleration regions, solute and solvent):

```
TITLE
GAMD by parts
END
GAMDATOMS
2
#   INATOM   FINATOM   AGROUP
         1       101        1
       102     22624        2
END
GAMDGROUPS
2
# GROUP_1   GROUP_2   ACCELGROUP
        1         1            1
        1         2            1
        2         2            2
END
```

Energy threshold and force constant for the potential energy term of the first acceleration region: -2775.959212 kJ/mol, 2.658963319 * 10^{-4} mol/kJ.
Energy threshold and force constant for the dihedral term of the first acceleration region: 308.2568275 kJ/mol, 6.679913200 * 10^{-3} mol/kJ.
Energy threshold and force constant for the potential energy term for the second acceleration region: -3.073428504 * 10^{5} kJ/mol, 2.394144099 * 10^{-5} mol/kJ.

Selective GaMD (one acceleration region for the Chignolin peptide only):

```
TITLE
GAMD by parts
END
GAMDATOMS
2
#   INATOM   FINATOM   AGROUP
         1       101        1
       102     22624        2
END
GAMDGROUPS
1
# GROUP_1   GROUP_2   ACCELGROUP
        1         1            1
        1         2            1
END
```

Energy threshold and force constant for the potential energy term: -2775.959212 kJ/mol, 2.658963319 * 10^{-4} mol/kJ.
Energy threshold and force constant for the dihedral term: 308.2568275 kJ/mol, 6.679913200 * 10^{-3} mol/kJ.

NaNaF N-Glycosylation

Acceleration groups, acceleration regions and parameters used for the different setups for the complex glycan NaNaF.

Standard GaMD:

```
TITLE
GAMD_all_atoms
END
GAMDATOMS
1
#   INATOM   FINATOM   AGROUP
         1     29672        1
END
GAMDGROUPS
1
# GROUP_1   GROUP_2   ACCELGROUP
        1         1            1
END
```

Energy threshold and force constant for the potential energy term: -4.011166404 * 10^{5} kJ/mol, 1.543508623 * 10^{-5} mol/kJ.
Energy threshold and force constant for the dihedral term: 1496.101265 kJ/mol, 2.913656304 * 10^{-3} mol/kJ.

Selective GaMD (two acceleration regions, solute and solvent):

```
TITLE
GAMD by parts
END
GAMDATOMS
2
#  INATOM   FINATOM   AGROUP
        1       227        1
      228     29672        2
END
GAMDGROUPS
2
# GROUP_1   GROUP_2   ACCELGROUP
        1         1            1
        1         2            1
        2         2            2
END
```

Energy threshold and force constant for the potential energy term of the first acceleration region: 436.4878857 kJ/mol, 2.806921170 * 10^{-4} mol/kJ.
Energy threshold and force constant for the dihedral term of the first acceleration region: 1504.160013 kJ/mol, 2.767872806 * 10^{-3} mol/kJ.
Energy threshold and force constant for the potential energy term for the second acceleration region: -4.010967613 * 10^{5} kJ/mol, 1.868235380 * 10^{-5} mol/kJ.

Selective GaMD (one acceleration region applied only to the complex glycan):

```
TITLE
GAMD by parts
END
GAMDATOMS
2
#  INATOM   FINATOM   AGROUP
        1       227        1
      228     29672        2
END
GAMDGROUPS
1
# GROUP_1   GROUP_2   ACCELGROUP
        1         1            1
        1         2            1
END
```

Energy threshold and force constant for the potential energy term: 436.4878857 kJ/mol, 2.806921170 * 10^{-4} mol/kJ.
Energy threshold and force constant for the dihedral term: 1504.160013 kJ/mol, 2.767872806 * 10^{-3} mol/kJ.

N-Glycosylated protein

Acceleration groups, acceleration regions and parameters used for the different setups for the N-glycosylated olfactory panda protein (5ngh) with a complex glycan NaNaF[2].

Standard GaMD:

```
TITLE
GAMD_all_atoms
END
GAMDATOMS
1
#   INATOM   FINATOM   AGROUP
         1     44565        1
END
GAMDGROUPS
1
# GROUP_1   GROUP_2   ACCELGROUP
         1         1            1
END
```

Energy threshold and force constant for the potential energy term: -6.167462140 * 10^5 kJ/mol, 1.178107799 * 10^{-5} mol/kJ.
Energy threshold and force constant for the dihedral term: 4461.436145 kJ/mol, 7.640953573 * 10^{-4} mol/kJ.

Selective GaMD (two acceleration regions, solute and solvent):

```
TITLE
GAMD by parts
END
GAMDATOMS
2
#   INATOM   FINATOM   AGROUP
         1      1938        1
      1939     44565        2
END
GAMDGROUPS
2
# GROUP_1   GROUP_2   ACCELGROUP
   1                   1                   1
   1                   2                   1
   2                   2                   2
END
```

Energy threshold and force constant for the potential energy term of the first acceleration region: -4.753364188 * 10^4 kJ/mol, 3.186494703 * 10^{-5} mol/kJ.
Energy threshold and force constant for the dihedral term of the first acceleration region: 4500.323829 kJ/mol, 6.657545714 * 10^{-4} mol/kJ.
Energy threshold and force constant for the potential energy term for the second acceleration region: -5.665988598 * 10^5 kJ/mol, 1.036376271 * 10^{-5} mol/kJ.

Selective GaMD (three acceleration regions, glycan, protein and solvent):

```
TITLE
GAMD by parts
END
GAMDATOMS
3
#   INATOM    FINATOM    AGROUP
         1       1728         1
      1729       1927         2
      1928       1938         1
      1939      44565         3
END
GAMDGROUPS
3
# GROUP_1    GROUP_2    ACCELGROUP
         1          1             1
         1          2             2
         1          3             1
         2          2             2
         2          3             2
         3          3             3
END
```

Energy threshold and force constant for the potential energy term of the first acceleration region: -4.799185614 * 10^{4} kJ/mol, 3.473753268 * 10^{-5} mol/kJ.
Energy threshold and force constant for the dihedral term of the first acceleration region: 3228.895191 kJ/mol, 8.486394752 * 10^{-4} mol/kJ.
Energy threshold and force constant for the potential energy term of the second acceleration region: 945.8113687 kJ/mol, 4.638631818 * 10^{-4} mol/kJ.
Energy threshold and force constant for the dihedral term of the first acceleration region: 1415.231799 kJ/mol, 3.8439219 * 10^{-3} mol/kJ.
Energy threshold and force constant for the potential energy term for the third acceleration region: -5.665988598 * 10^{5} kJ/mol, 1.048809273 * 10^{-5} mol/kJ.

References

(1) Hansen, N.; Öehlknecht, C.; de Ruiter, A.; Lier, B.; van Gunsteren, W. F.; Oostenbrink, C.; Gebhardt, J. A Suite of Advanced Tutorials for the GROMOS Biomolecular Simulation Software [Article v1.0]. *Living J Comput Mol Sci* **2020**, *2* (1). https://doi.org/10.33011/livecoms.2.1.18552.

(2) Zhu, J.; Arena, S.; Spinelli, S.; Liu, D.; Zhang, G.; Wei, R.; Cambillau, C.; Scaloni, A.; Wang, G.; Pelosi, P. Reverse Chemical Ecology: Olfactory Proteins from the Giant Panda and Their Interactions with Putative Pheromones and Bamboo Volatiles. *Proceedings of the National Academy of Sciences* **2017**, *114* (46). https://doi.org/10.1073/pnas.1711437114.
